# Multi-omics analysis of soy isoflavone-induced responses in rumen fermentation, endocrine status and milk production in cows with varying milk yields

**DOI:** 10.1186/s40104-026-01397-9

**Published:** 2026-05-08

**Authors:** Xingwei Jiang, Chenguang Zhang, Yuhao Zhang, Jing Li, Jianrong Ren, Jiarui Wang, Xinfeng Hou, Zhihong Zhang, Shengru Wu, Junhu Yao

**Affiliations:** 1 College of Animal Science and Technology, Northwest A&F University, Yangling, Shaanxi 712100 China; 2 National Center of Technology Innovation for Dairy, Hohhot, 010100 China; 3 Leyuan Animal Husbandry, JUNLEBAO Company, Shijiazhuang, Hebei 050221 China

**Keywords:** Cow, Lactation performance, Microbiota, Rumen, Soy isoflavones

## Abstract

**Background:**

Improving milk yield and feed efficiency is pivotal for climate-smart dairy systems, as rumen mediated fermentation governs energy and nitrogen utilization and thereby greenhouse-gas emission intensity. Soybean isoflavones (SIF) may modulate rumen fermentation, yet their effects on rumen function, microbiome features, host endocrine/metabolic responses, and lactation performance-particularly across cows with divergent milk-yield phenotypes-remain unclear.

**Results:**

Fifty‑six lactating Holstein cows (28 high‑yield cows, HY; 28 low‑yield cows, LY) were divided into two categories by milk yield. Within each yield category, cows were randomly assigned to one of two dietary treatments: a basal diet (Control) or the basal diet supplemented with SIF at 0.01% of dry matter. This yielded a 2 × 2 factorial design with four experimental groups (*n* = 14 per group): high‑yield control (HCON), high‑yield SIF (HSIF), low‑yield control (LCON), and low‑yield SIF (LSIF). SIF increased milk yield by 8.75% and improved fat-corrected milk (+ 7.20%), dry matter intake (+ 3.20%), and feed efficiency (+ 3.26%), with larger gains in HY cows (milk yield + 8.89%; feed efficiency + 4.55%). Rumen fermentation shifted toward a more energetically favorable profile, with lower acetate (– 2.70%), higher propionate (+ 4.55%), and a reduced acetate-to-propionate ratio (– 7.02%), accompanied by increased microbial crude protein (+ 21.53%) without changes in pH or NH_3_-N. SIF altered endocrine status irrespective of phenotype, increasing estradiol and progesterone while decreasing prolactin and growth hormone, and reduced blood ALP, lactate, and triglycerides. Metagenomics indicated phenotype-dependent microbial and functional responses to SIF: HY cows showed enrichment of taxa (e.g., *Caudoviricetes* sp*., Eubacterium* sp*.,* and *Butyrivibrio* sp*.*) associated with amino-acid, cofactor metabolism and propionate pathways, whereas LY cows exhibited enrichment of *Prevotella* sp*.* and *Bacteroides* sp*.* with functions favoring carbohydrate degradation. The HCON group exhibited greater abundances of *Prevotella* sp*.* and *Hallella* spp*.* with enhanced carbohydrate degradation functions, whereas the LCON group was enriched in *Ruminococcus* sp*.* and *Methanobrevibacter* sp*.*, associated with methane metabolism.

**Conclusions:**

In conclusion, this study highlights the potential of SIF supplementation to improve lactation efficiency, modulate rumen microecology and endocrine function in dairy cows. These findings establish a theoretical framework for achieving efficient and precise feeding management on large-scale dairy farms.

## Background

Owing to the growing global population, increasing competition for resources, and the rising environmental pressures associated with dairy production, improving milk yield and feed efficiency has become a key objective in the pursuit of dairy systems that limit greenhouse gas emissions and support more sustainable production [1, 2]. Improving feed conversion efficiency not only reduces the carbon and nitrogen intensity of milk but also aligns with the goals of climate-smart agriculture, whereby reshaping rumen fermentation toward energetically favorable pathways through nutritional intervention can mitigate methane emissions and enhance nutrient utilization [3, 4]. Besides directly modulating the rumen, silage inoculants have been reported to influence silage quality, rumen fermentation and microbial community structure, methane emissions, and milk yield, thereby underscoring the central role of rumen processes in determining feed‑use efficiency [5]. The rumen hosts diverse communities of microorganisms, including bacteria, archaea, protozoa, fungi and viruses. These microorganisms establish a close symbiotic relationship with their ruminant hosts [6–8]. Through feed degradation and nutrient synthesis, rumen microbial dynamics are primarily responsible for providing nutrients to the host [9–12]. Dietary nutrients are degraded by rumen microbes into volatile fatty acids (VFA, mainly acetate, propionate, and butyrate), adenosine triphosphate (ATP), carbon dioxide, methane, and ammonia [13]. ATP primarily supports microbial maintenance and growth, whereas VFA and microbial biomass provide energy and protein to the ruminant host [14, 15]. Accordingly, microbially mediated rumen fermentation determines the health, growth, and production performance of the host.

Soybean isoflavones (SIF) are naturally occurring flavonoids found in leguminous plants [16]. SIF are widely present in corn-soybean-based animal diets. Owing to their structural similarity to mammalian estrogens and their estrogenic activity in animals, SIF are classified as phytoestrogens. Daidzin and genistin are the two predominant glycosylated forms of SIF [17]. Glycosylated isoflavones cannot be directly absorbed by the intestine. The hydrolysis of these compounds into their corresponding aglycones is the first and most critical step in the generation of biological activity during isoflavone metabolism. This hydrolysis (deglycosylation) relies on β-glucosidases, which are secreted by intestinal microbes such as *Bacteroides*, *Bifidobacterium*, and *Lactobacillus* [18]. Within the intestinal lumen, daidzein and genistein undergo deglycosylation, dehydroxylation, reduction, C-ring cleavage, and demethylation [19], resulting in final metabolites such as equol and p-ethylphenol [17, 20]. SIF have various biological activities, including antioxidant, anti-inflammatory, and differentiation-inducing effects, influencing physiological and metabolic processes in many animals [21, 22]. In addition, SIF can also enhance the immune function of cows [23] and pigs [24], the nitrogen metabolism of dairy cows [25], the intestinal integrity and meat quality of pigs [26], and the secretion of metabolic hormones in poultry [27, 28].

In vitro fermentation assays showed that propionate production exhibited a quadratic response to increasing doses of SIF (0, 5, 10, 20, 50, 100, and 200 mg/L), concomitant with enhanced fermentative activity of ruminal fungi. These results suggest that SIF can modulate the rumen microbiota, although the underlying mechanisms remain unclear [29]. Another study reported that cow rumen fluid can degrade SIF to produce the metabolite equol [30]. Multiple bacterial taxa, including *Ruminococcus*, *Bacteroides*, *Bifidobacterium*, and *Eubacterium*, have been implicated in the hydrolysis of isoflavone glycosides [31, 32], and these species are widely distributed in the rumen [33]. A dietary supplementation trial in beef cattle reported that SIF increased the relative abundances of *Prevotella*, *RC9_gut_group*, *Succinivibrio*, and *Ruminobacter*, which was associated with increased VFA production and enhanced microbial crude protein (MCP) synthesis, indicative of improved rumen function [6]. Moreover, differences in rumen metabolism and microbial community composition at comparable physiological stages have been associated with variations in dairy cow production performance [34–36]. Although existing studies indicate that SIF may affect ruminant production and rumen fermentation, there is a paucity of research that systematically integrates rumen fermentation, microbiome functional potential, endocrine responses, and production performance across cows with differing milk-yield levels. Therefore, this study aims to compare responses to SIF between high- and low-yielding dairy cows, monitor changes in key hormonal profiles, and integrate metagenomic and metabolomic data to systematically elucidate the links among SIF, the rumen microbiome and its functions, and feed utilization efficiency. We hypothesize that SIF can improve energy utilization and lactation performance by reshaping the rumen microbiome—altering fermentation patterns and key metabolic pathways—and by modulating host endocrine status, and that these effects may be more pronounced in high-yield cows.

## Materials and methods

### Animals, diets, and experimental design

The experiment was conducted at Junlebao Qizhi Dairy Farm (Hebei, China). We stratified milk-yield phenotypes using an unsupervised K-means clustering approach in a cohort of 400 primiparous Holstein cows maintained under comparable diets and management. Cows with comparable diets, management, 7‑day average milk yield (33.29 ± 8.21 kg/d), days in milk (50.73 ± 9.40 d) and body condition score (3.11 ± 0.18). Iterative K-means clustering identified two well-separated clusters, with centroid milk yields of approximately 40.86 kg/d and 25.29 kg/d, respectively, showing a significant gap of about 15.57 kg/d between them. Additionally, based on a priori power analysis, under the conditions of α = 0.05 and power = 0.80, the minimum sample size required to detect a medium effect size (Cohen's *d* = 0.80) was approximately 25. Subsequently, cows with health abnormalities (based on clinical records or abnormal body temperature) were excluded. Under the aforementioned conditions, this study selected 28 high-yield (41.50 ± 0.61 kg/d) and 28 low-yield (25.08 ± 0.91 kg/d) cows, which not only met the requirement for detecting a medium effect size but also retained sufficient samples (*n* = 14) for subsequent subgroup analysis. Therefore, the cutoff of about 16.42 kg/d (> 15.57 kg/d) reflects the natural stratification of milk yield capacity within the population rather than a subjectively defined value, and it has solid statistical as well as biological justification. The high-yield and low-yield cows were each randomly divided into two groups, resulting in four treatments: high-yield control group (HCON), low-yield control group (LCON), high- and low-yield groups supplementing SIF (HSIF and LSIF), respectively. The control groups were fed the basal diet (Table 1), whereas the SIF groups received the same basal diet supplemented with 0.01%DM of SIF. The dosage was based on previous reports [37, 38] and the manufacturer’s guidelines. The SIF product (Fengpeng Biotechnology Co., Ltd., Guilin, China) contained 9.02% daidzin, 0.43% glycitin, 38.96% genistin, and 51.6% defatted rice bran as a carrier and was premixed with feed before use. All cows were individually housed in adjacent pens for 35 d. They were fed a total mixed ration (TMR) three times daily at 08:00, 15:00 and 22:00, with ad libitum access to feed and water. Each pen was equipped with an individual feed trough to record daily feed intake.

**Table 1:** Ingredients and nutrient composition of the basal diet (dry matter basis)

Items	Content
Ingredients, %	
Corn silage	41.17
Alfalfa haylage	17.00
Corn grain	10.66
Flaking corn	8.24
Soybean meal	9.21
Rumen-protected soybean meal	1.21
Roasted soybeans	1.45
Canola meal	1.21
Beet granules	3.64
Whole cottonseed	3.88
Premix^1^	1.80
NaHCO_3_	0.53
Chemical composition^2^, g/kg	
CP	187.10
Starch	291.90
NDF	230.60
ADF	171.30
Ash	67.30
Ca	10.50
P	5.20

^1^Contained (concentration of premix per kilogram of DM): 132 g of chloride, 65 g of magnesium, 50 g of phosphorus, 90 g of sodium, 1.5 g of potassium, 0.5 g of sulfur, 4,500 mg of zinc, 4,000 mg of manganese, 1,500 mg of copper, 225 mg of iodine, 50 mg of selenium, 25 mg of cobalt, 600,000 IU of vitamin A, 190,000 IU of vitamin D, and 4,000 IU of vitamin E

^2^*CP* Crude protein, *NDF* Neutral detergent fibre, *ADF* Acid detergent fibre

### Feed sample collection and nutritional component analysis

Feed samples were collected weekly from the beginning, middle, and end of TMR discharge, thoroughly mixed, oven-dried at 65 °C for 24 h, ground through a 1-mm sieve, and stored at −20 °C for analysis. Dry matter (DM) and crude protein (CP) were determined according to the AOAC method [39], neutral detergent fibre (NDF) and acid detergent fibre (ADF) were analyzed via method of Van Soest et al. [40], and starch content was measured via the ISO 6493:2000 method.

### Milk sample collection, milk composition analysis and rumination time record

The milk yield was automatically recorded three times daily at 08:00, 15:00 and 22:00 via a DeLaval Rotary E500 milking system (Sweden). The average milk yield from 0–35 d was calculated for each cow. Milk composition was determined from samples collected on d 33–35 (3 d mean). At each of the three daily milkings, 50 mL milk was collected per cow using a milk sampler, pooled within day at a 4:3:3 ratio (morning:midday:evening), mixed, and analyzed on the sampling day (MilkoScan™ FT3, Denmark) for fat, protein, lactose, and solids-not-fat. Ruminating time (RT) was automatically recorded daily using smart collars (AfiCollar, Israel) and the farm management system (DelPro™, DeLaval, Sweden), and the average from 0–35 d was calculated for each cow. The fat-corrected milk yield (FCM) was calculated as follows [41]:

$$\mathrm{FCM }= 0.4 \times \mathrm{ milk\;yield }(\mathrm{kg}/\mathrm{d}) + 15 \times \mathrm{ milk\;fat\;yield }(\mathrm{kg}/\mathrm{d}).$$

### Blood sample collection and biochemical parameter and hormones analysis

On d 35, blood samples were collected from the tail vein before morning feeding into EDTA-2K vacuum tubes (Yinuo Biotechnology Co., Ltd., Jiangxi, China). The plasma was separated via centrifugation (1,000 × *g*, 15 min, 4 °C; 5424R, Eppendorf, Germany) and stored at −20 °C. The biochemical parameters included alkaline phosphatase (ALP), alanine aminotransferase (ALT), aspartate aminotransferase (AST), lactate dehydrogenase (LDH), and superoxide dismutase (SOD) were analysed via an automatic biochemical analyser (Hitachi 7600, Tokyo, Japan) and albumin (ALB), cholesterol (CHO), glucose (GLU), lactic acid (LAC), total bile acid (TBA), triglyceride (TG), urea nitrogen (UREA), and total protein (TP) were analysed via a chemiluminescence analyser (Autolumo A2000, Zhengzhou, China). The levels of plasma estradiol (E2), prolactin (PRL), progesterone (PROG), growth hormone (GH), and insulin (INS) were determined via commercial ELISA kits (Enzyme-linked Biotechnology Co., Ltd., Shanghai, China).

### Rumen fluid collection and ruminal VFA, MCP, NH₃-N content analysis

Rumen fluid was collected via an oral stomach tube before the morning feeding on d 35. After filtration through four layers of cheesecloth, samples were successfully obtained and aliquoted from 56 cows (HCON, LCON, HSIF, LSIF, *n* = 14). Samples allocated for metagenomic and metabolomic analyses were stored at −80 °C, while aliquots designated for fermentation parameter determination were stored at −20 °C and analyzed within one month. VFA were quantified via an Agilent 6850 gas chromatograph (Agilent Technologies, CA, USA) equipped with a HP-FFAP column (30 m × 0.25 mm, 0.25 μm) and a flame ionization detector, as in our previous studies [42, 43]. MCP was determined via differential centrifugation. Briefly, thawed rumen fluid samples were centrifuged at 1,000 × *g* for 10 min. Two milliliters of the resulting supernatant were transferred and centrifuged at 15,996 × *g* for 20 min to pellet the microbial fraction. The supernatant was discarded, and the pellet was washed twice by resuspension in 1.5 mL of 0.9% saline followed by centrifugation at 15,996 × *g* for 35 min each. The final pellet was carefully resuspended in a small volume of distilled water and transferred to a centrifuge tube for storage until analysis. Protein concentration was measured using a commercial BCA protein assay kit (A045-4, Nanjing Jiancheng Bioengineering Institute, China) following the manufacturer’s instructions. A standard curve was prepared as instructed, and samples were diluted to fall within the assay’s detection range prior to quantification. Filtered rumen fluid was centrifuged (8,161 × *g* for 10 min at 4 °C) to obtain a clear supernatant, which was further analyzed for ammonia nitrogen (NH₃-N) content using a phenol-hypochlorite assay [44].

### Rumen fluid metagenome sequencing and analysis

Microbial DNA from the rumen fluid samples was extracted from 56 cows using the E.Z.N.A. A DNA kit (Omega Biotek, Norcross, GA, USA) according to the manufacturer’s protocol. DNA fragments (~ 350 bp) were used to construct paired-end libraries (Bioo Scientific, TX, USA), which were subsequently sequenced on an Illumina NovaSeq X Plus platform. The raw reads were processed via fastp [45] (https://github.com/OpenGene/fastp, v0.20.0), assembled via MEGAHIT [46] (https://github.com/voutcn/megahit, version 1.1.2), and clustered via CD-HIT [47] (http://weizhongli-lab.org/cd-hit/, version 4.7) to generate nonredundant gene sets. Alpha diversity is applied in analyzing complexity of species diversity for a sample through Shannon index, and Beta diversity was calculated based on Bray–Curtis distance matrices. Taxonomic and functional annotations were performed via DIAMOND [48] (https://github.com/bbuchfink/diamond, version 2.0.13) against the NR and KEGG databases (e-value ≤ 1e-5), and carbohydrate-active enzymes were identified via HMMER (http://hmmer.org/, version 3.1b2) against the CAZy database.

### Rumen fluid metabolomic sequencing and analysis

The samples of rumen fluid from 56 cows were used for metabolomics analysis. Briefly, 100 µL of thawed rumen fluid was extracted with 400 µL of acetonitrile–methanol (1:1) containing internal standards. The supernatant was dried under nitrogen and redissolved for LC–MS analysis via a UHPLC-Orbitrap Exploris 240 system (Thermo Fisher Scientific, USA). The raw data were processed with Progenesis QI (Waters Corp., USA) for peak alignment and metabolite identification via HMDB (http://www.hmdb.ca/) and Metlin databases (https://metlin.scripps.edu/). Statistical analyses, including PCA and OPLS-DA (using ropls v1.6.2 in R), were performed to identify differentially abundant metabolites (VIP > 1, *P* < 0.05), and KEGG pathway enrichment was conducted via scipy.stats (Python package).

## Statistical analysis

The data were analysed via SAS 9.4 (SAS Institute Inc., Cary, NC, USA). The UNIVARIATE procedure was used to examine the normality and detect outliers in the cow phenotypic data (including production performance, rumen fermentation parameters, blood biochemical indices and hormones). Two-way ANOVA (PROC GLM) was performed via the GLM procedure to evaluate the effects of SIF supplementation (with vs. without), milk yield level (high vs. low), and their interaction on milk yield and composition, FCM, rumination time, dry matter intake (DMI), feed efficiency, rumen fermentation parameters, blood biochemical indices, and hormone levels. When a significant main effect or interaction (*P* < 0.05) was observed, Tukey’s multiple comparison test was applied to compare least squares means between HCON and HSIF, and between LCON and LSIF. The *t*-test was performed to evaluate the effects of SIF supplementation on production performance, ruminal fermentation parameters, blood biochemical indices, and hormone profiles in cows with the same milk production level (high or low-yield). The results are expressed as least squares means. Differences were considered significant at *P* < 0.05 and as a tendency at 0.05 ≤ *P* < 0.10.

For the rumen microbial data, the Wilcoxon rank-sum test, Kruskal–Wallis H test, and linear discriminant analysis effect size (LEfSe) were used to identify differences in microbial taxa, KEGG pathways and enzymes, and CAZymes. Features with a false discovery rate (FDR)-adjusted *P* value < 0.05 were considered significantly different. Rumen metabolomic data were compared via the Wilcoxon rank-sum test, and metabolites with FDR-adjusted *P* < 0.05 and variable importance in projection (VIP) > 1 from orthogonal partial least squares discriminant analysis (OPLS-DA) were regarded as significantly different.

Spearman’s correlation was performed to assess the relationships between differential microbial taxa and production performance, between performance and rumen fermentation or hormone levels, and between differential microbes and metabolites. Correlations with |*r*| > 0.5 and *P* < 0.05 were considered significant. Correlation matrices of differential microbial data were constructed via Spearman’s correlation, and Mantel tests based on Euclidean distances were conducted to validate associations between differential microbial profiles and KEGG pathway enrichment of metabolites. Data visualization and correlation mapping were conducted via the Majorbio Cloud Platform (www.majorbio.com).

## Results

### Production responses to SIF supplementation in high- and low-yield cows

The differential effect of supplementing SIF on the production performance of high-yield and low-yield dairy cows is shown in Table 2. SIF significantly increased milk yield by 8.75%, as well as yields of milk components: fat + 6.12%, SNF + 8.03%, protein + 7.62% and lactose + 7.84%. SIF also increased FCM by 7.20%, DMI by 3.20% and feed efficiency by 3.26% (*P*_Treat_ < 0.05). However, SIF did not significantly alter milk fat, SNF, protein, lactose percentages, or rumination time (*P*_Treat_ > 0.05). The enhancing effect of SIF on milk yield was more pronounced in high-yield cows (increase 8.89%; *P*_HY-CON vs. HY-SIF_ = 0.017), whereas the effect was not significant in low-yield cows (*P*_LY-CON vs. LY-SIF_ = 0.308). In high-yield cows, SIF significantly increased the yields of milk fat (+ 9.04%), SNF (+ 8.52%), protein (+ 7.91%), lactose (+ 8.54%), FCM (+ 8.92%), DMI (+ 3.63%), and feed efficiency (+ 4.55%) (*P*_HY-CON vs. HY-SIF_ < 0.05). No comparable effects were observed in low-yield cows (*P*_LY-CON vs. LY-SIF_ > 0.05). The interaction effect between SIF supplementation and milk production level was not significant for DMI, feed efficiency, RT, and lactation performance (*P*_Treat × Produc_ > 0.05). The results of the main-effect analyses indicated a greater improvement in lactational performance in the high-yield cows.

**Table 2:** Differential responses of DMI, feed efficiency, RT, and lactation performance to SIF supplementation in high- and low-yield dairy cows

Treatment^1^	CON		SIF		SEM	*P* value				
Production	HY	LY	HY	LY		*P*_Treat_	*P*_Produc_	*P*_Treat × Produc_	*P*_HY-CON vs. HY-SIF_	*P*_LY-CON vs. LY-SIF_
DMI, kg/d	26.69	21.41	27.66	21.98	0.316	0.042	< 0.001	0.900	0.024	0.589
Feed efficiency	1.54	1.22	1.61	1.24	0.018	0.013	< 0.001	0.561	0.028	0.626
milk yield, kg/d	40.25	24.12	43.83	26.17	0.877	0.003	< 0.001	0.721	0.017	0.308
Fat, %	4.14	4.76	4.12	4.36	0.065	0.376	0.002	0.240	0.830	0.206
SNF, %	9.06	9.15	9.00	9.04	0.028	0.203	0.297	0.869	0.570	0.253
Protein, %	3.45	3.49	3.43	3.45	0.011	0.260	0.136	0.851	0.641	0.279
Lactose, %	4.96	4.99	4.93	4.91	0.019	0.241	0.084	0.783	0.640	0.169
Fat yield, kg/d	1.66	1.12	1.81	1.14	0.036	0.036	< 0.001	0.522	0.043	0.810
SNF yield, kg/d	3.64	2.21	3.95	2.37	0.079	0.008	< 0.001	0.736	0.027	0.385
Protein yield, kg/d	1.39	0.84	1.50	0.90	0.030	0.008	< 0.001	0.743	0.030	0.387
Lactose yield, kg/d	1.99	1.20	2.16	1.28	0.043	0.008	< 0.001	0.732	0.027	0.405
FCM, kg/d	41.04	26.43	44.70	27.63	0.871	0.011	< 0.001	0.587	0.025	0.589
RT, min/d	440.03	405.03	438.29	397.65	4.876	0.729	0.006	0.953	0.904	0.667

*DMI* Dry matter intake, *SNF* Solids-not-fat, *FCM* Fat-corrected milk yield, *RT* Rumination time

^1^ HCON, LCON, HSIF, LSIF (*n* = 14), *P*_Treat_: with vs. without SIF, *P*_Produc_: high- vs. low-yield

### Rumen fermentation shifts after SIF supplementation

The differential responses of the rumen fermentation parameters of high-yield and low-yield cows to SIF supplementation are presented in Table 3. SIF tended to increase the propionate concentration (*P*_Treat_ = 0.060) but did not affect the concentrations of acetate, isobutyrate, butyrate, isovalerate, valerate, or total VFA (*P*_Treat_ > 0.05). In molar proportions, SIF decreased acetate by 2.70% (*P*_Treat_ = 0.008), increased propionate by 4.55% (*P*_Treat_ = 0.007), lowered A:P by 7.02% (*P*_Treat_ = 0.006), and elevated MCP by 21.53% (*P*_Treat_ = 0.0071), with no effect on NH_3_-N or pH (*P*_Treat_ > 0.05). There was a significant interaction effect between treatment and production for isobutyrate (*P*_Treat × Produc_ = 0.036). In high-yield cows, SIF tended to increase the propionate concentration and proportion and lower the A:P ratio (0.05 ≤ *P*_HY-CON vs. HY-SIF_ < 0.10). In low-yield cows, SIF tended to increase the isovalerate concentration and proportion and reduce the acetate proportion (0.05 ≤ *P*_LY-CON vs. LY-SIF_ < 0.10). SIF significantly increased MCP in both high-yield (+ 24.25%) and low-yield cows (+ 18.88%) (*P* < 0.05).

**Table 3:** Differential responses of rumen fermentation parameters to SIF supplementation in high- and low-yield dairy cows

Treatment^1^	CON		SIF		SEM	*P* value				
Production	HY	LY	HY	LY		*P*_Treat_	*P*_Produc_	*P*_Treat × Produc_	*P*_HY-CON vs. HY-SIF_	*P*_LY-CON vs. LY-SIF_
Concentration, mmol/L										
Acetate	43.97	46.93	46.65	46.69	0.790	0.881	0.487	0.518	0.385	0.927
propionate	22.81	21.79	26.42	23.08	0.551	0.060	0.241	0.610	0.077	0.458
Isobutyrate	0.60	0.58	0.58	0.67	0.015	0.685	0.474	0.196	0.725	0.108
Butyrate	7.15	7.87	8.17	8.30	0.162	0.414	0.533	0.103	0.111	0.454
Isovalerate	1.11	1.07	1.14	1.26	0.030	0.333	0.603	0.326	0.844	0.079
Valerate	1.32	1.22	1.38	1.18	0.033	0.775	0.111	0.666	0.670	0.741
TVFA	76.96	79.45	84.34	81.19	1.429	0.369	0.815	0.471	0.195	0.711
Percentage, %										
Acetate	57.13	59.11	55.46	57.64	0.298	0.008	0.009	0.977	0.104	0.081
propionate	29.63	27.31	31.20	28.33	0.318	0.007	0.002	0.700	0.083	0.289
Isobutyrate	0.78	0.73	0.69	0.83	0.016	0.811	0.191	0.036	0.108	0.100
Butyrate	9.29	9.97	9.65	10.19	0.108	0.816	0.017	0.065	0.190	0.643
Isovalerate	1.44	1.34	1.36	1.56	0.032	0.591	0.286	0.144	0.524	0.055
Valerate	1.73	1.54	1.63	1.46	0.031	0.188	0.009	0.970	0.477	0.429
A:P	1.95	2.18	1.79	2.05	0.033	0.006	0.005	0.844	0.069	0.206
NH_3_-N, mg/dL	14.24	15.28	14.49	14.93	0.136	0.676	0.094	0.624	0.489	0.606
pH	6.29	6.37	6.27	6.31	0.028	0.408	0.630	0.892	0.872	0.590
MCP, μg/mL	7,249.97	6,154.07	9,007.93	7,282.32	225.775	0.001	0.025	0.825	0.037	0.041

*TVFA* Total volatile fatty acid, *A:P* The ratio of acetate to propionate, *MCP* Microbial crude protein

^1^HCON, LCON, HSIF, LSIF (*n* = 14), *P*_Treat_: with vs. without SIF, *P*_Produc_: high- vs. low-yield

### Blood biochemical indices and hormones responses to SIF supplementation in high- and low-yield cows

The differential responses of blood biochemical indices of high-yield and low-yield cows to SIF supplementation are shown in Table 4. SIF significantly decreased ALP, LAC, and TG (*P*_Treat_ < 0.05) but did not affect ALB, ALT, AST, CHO, GLU, LDH, TBA, UREA, TP, or SOD (*P*_Treat_ > 0.05). There was no significant interaction effect between treatment and production for all the blood biochemical indices (*P*_Treat × Produc_ > 0.05). Further analysis revealed higher GLU and lower LAC in HSIF vs. HCON (*P*_HY-CON vs. HY-SIF_ < 0.05) and a trend toward lower LAC in LSIF vs. LCON (*P*_LY-CON vs. LY-SIF_ = 0.052).

**Table 4:** Differential responses of blood biochemical indices to SIF supplementation in high- and low-yield dairy cows

Treatment^1^	CON		SIF		SEM	*P* value				
Production	HY	LY	HY	LY		*P*_Treat_	*P*_Produc_	*P*_Treat × Produc_	*P*_HY-CON vs. HY-SIF_	*P*_LY-CON vs. LY-SIF_
ALB, g/L	20.15	20.76	21.29	20.19	0.307	0.922	0.915	0.472	0.292	0.613
ALP, U/L	6.08	4.20	4.86	4.50	0.235	0.029	0.126	0.107	0.185	0.646
ALT, U/L	22.89	21.91	20.19	21.91	0.614	0.697	0.886	0.419	0.130	0.999
AST, U/L	75.92	83.35	78.81	71.87	2.591	0.571	0.902	0.526	0.573	0.297
CHO, mmol/L	4.57	3.94	4.67	4.06	0.120	0.983	0.094	0.844	0.787	0.821
GLU, mmol/L	3.01	3.48	3.36	3.53	0.045	0.107	0.007	0.169	0.015	0.787
LAC, mg/dL	13.62	12.17	9.60	10.35	0.378	0.006	0.893	0.121	0.007	0.052
LDH, U/L	736.46	817.78	745.07	832.93	14.603	0.936	0.073	0.902	0.771	0.845
TBA, umol/L	184.29	133.68	176.76	126.82	8.799	0.475	0.039	0.907	0.801	0.877
TG, mmol/L	0.11	0.11	0.08	0.08	0.004	0.002	0.919	0.954	0.096	0.109
UREA, mmol/L	4.81	4.65	4.60	4.43	0.092	0.502	0.658	0.813	0.484	0.772
TP, g/L	54.28	63.59	59.41	66.12	1.218	0.342	0.002	0.537	0.112	0.622
SOD, U/L	132.31	129.22	143.79	124.71	2.327	0.910	0.082	0.165	0.131	0.773

*ALB* Albumin, *ALP* Alkaline phosphatase, *ALT* Alanine aminotransferase, *AST* Aspartate aminotransferase, *CHO* Cholesterol, *GLU* Glucose, *LAC* Lactic acid, *LDH* Lactate dehydrogenase, *TBA* Total bile acid, *TG* Triglyceride, *UREA* Urea nitrogen, *TP* Total protein, *SOD* Superoxide dismutase

^1^HCON, LCON, HSIF, LSIF (*n* = 14), *P*_Treat_: with vs. without SIF, *P*_Produc_: high- vs. low-yield

With respect to hormones (Table 5), SIF increased E2 and PROG and decreased PRL and GH (*P*_Treat_ < 0.001), with no effect on INS (*P*_Treat_ > 0.05). No interaction effect between treatment and production was detected for the aforementioned parameters (*P*_Treat × Produc_ > 0.05). The same hormonal responses were observed in both high-yield and low-yield dairy cows, indicating that the regulation of cow endocrine function by SIF does not depend on milk production levels.

**Table 5:** Differential responses of blood hormone level to SIF supplementation in high- and low-yield dairy cows

Treatment^1^	CON		SIF		SEM	*P* value				
Production	HY	LY	HY	LY		*P*_Treat_	*P*_Produc_	*P*_Treat × Produc_	*P*_HY-CON vs. HY-SIF_	*P*_LY-CON vs. LY-SIF_
E2, pg/mL	199.71	212.77	225.29	247.11	2.983	< 0.001	< 0.001	0.682	0.001	< 0.001
RPL, mIU/L	914.83	858.88	822.87	776.44	9.595	< 0.001	< 0.001	0.714	0.001	0.003
PROG, ng/mL	2.53	2.74	2.98	3.17	0.043	< 0.001	< 0.001	0.753	< 0.001	< 0.001
GH, ng/mL	17.60	16.31	15.91	14.55	0.180	< 0.001	< 0.001	0.981	< 0.001	< 0.001
INS, mIU/L	39.47	38.78	36.19	37.55	0.531	0.477	0.836	0.102	0.054	0.582

*E2* Estradiol, *PRL* Prolactin, *PROG* Progesterone, *GH* Growth hormone, *INS* Insulin

^1^ HCON, LCON, HSIF, LSIF (*n* = 14), *P*_Treat_: with vs. without SIF, *P*_Produc_: high- vs. low-yield

### Rumen microbiome structure, function, and phenotype correlations in high- and low-yield cows

**Fig. 1:**
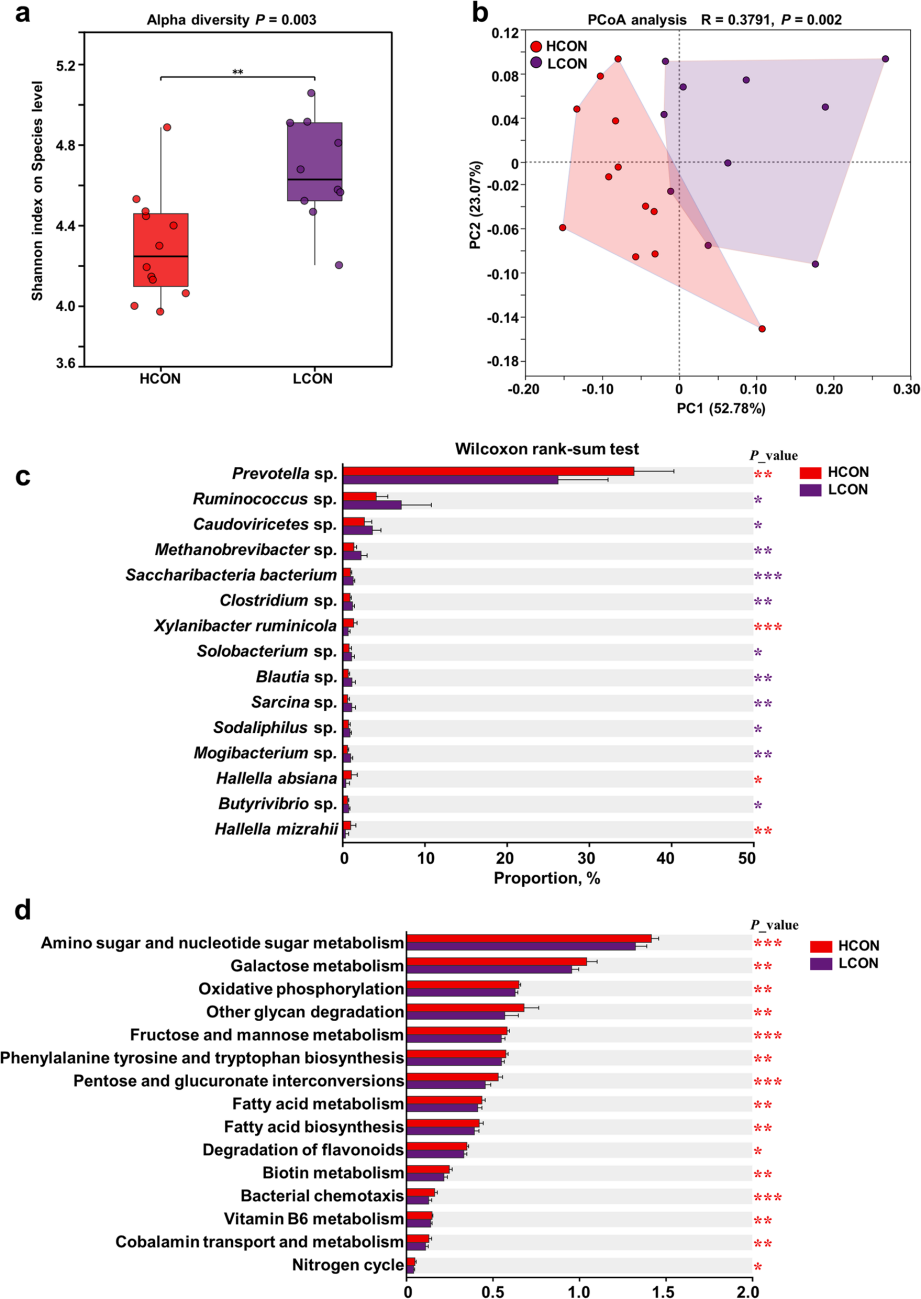
Differences in rumen microbial community structure and function between high- and low-yield dairy cows. **a** Shannon index (α diversity). **b** PCoA of Bray–Curtis distances (PC1 = 52.78%, PC2 = 20.27%; PERMANOVA *R* = 0.3791, *P* = 0.002). **c** Top 15 differential species (Wilcoxon rank-sum test, *P* < 0.05). **d** KEGG pathway enrichment (*P* < 0.05)

The differences in the rumen microbial composition and functions between high-yield and low-yield cows were further identified (Fig. 1). Shannon diversity was lower in HCON than in LCON (*P* = 0.003). PCoA revealed clear separation between groups (PC1 = 52.78%, PC2 = 20.27%; *R* = 0.3791, *P* = 0.002), indicating significant differences in β diversity. The species enriched in HCON included *Prevotella* sp.*, Xylanibacter ruminicola, Hallella absiana*, and *Hallella mizrahii*, whereas *Ruminococcus* sp.*, Caudoviricetes* sp.*, Methanobrevibacter* sp.*, Saccharibacteria bacterium, Clostridium* sp.*, Solobacterium* sp.*, Blautia* sp.*, Sarcina* sp.*, Sodaliphilus* sp.*, Mogibacterium* sp.*,* and *Butyrivibrio* sp. were more abundant in LCON (relative abundance Top 15; *P* < 0.05, Fig. 1c). KEGG analyses revealed greater capacities of HCON for amino sugar and nucleotide sugar metabolism, galactose metabolism, oxidative phosphorylation, other glycan degradation, fructose and mannose metabolism, phenylalanine tyrosine and tryptophan biosynthesis, pentose and glucuronate interconversions, fatty acid metabolism, fatty acid biosynthesis, flavonoid degradation, biotin metabolism, bacterial chemotaxis, vitamin B_6_ metabolism, cobalamin transport and metabolism and the nitrogen cycle (*P* < 0.05, Fig. 1d).

**Fig. 2:**
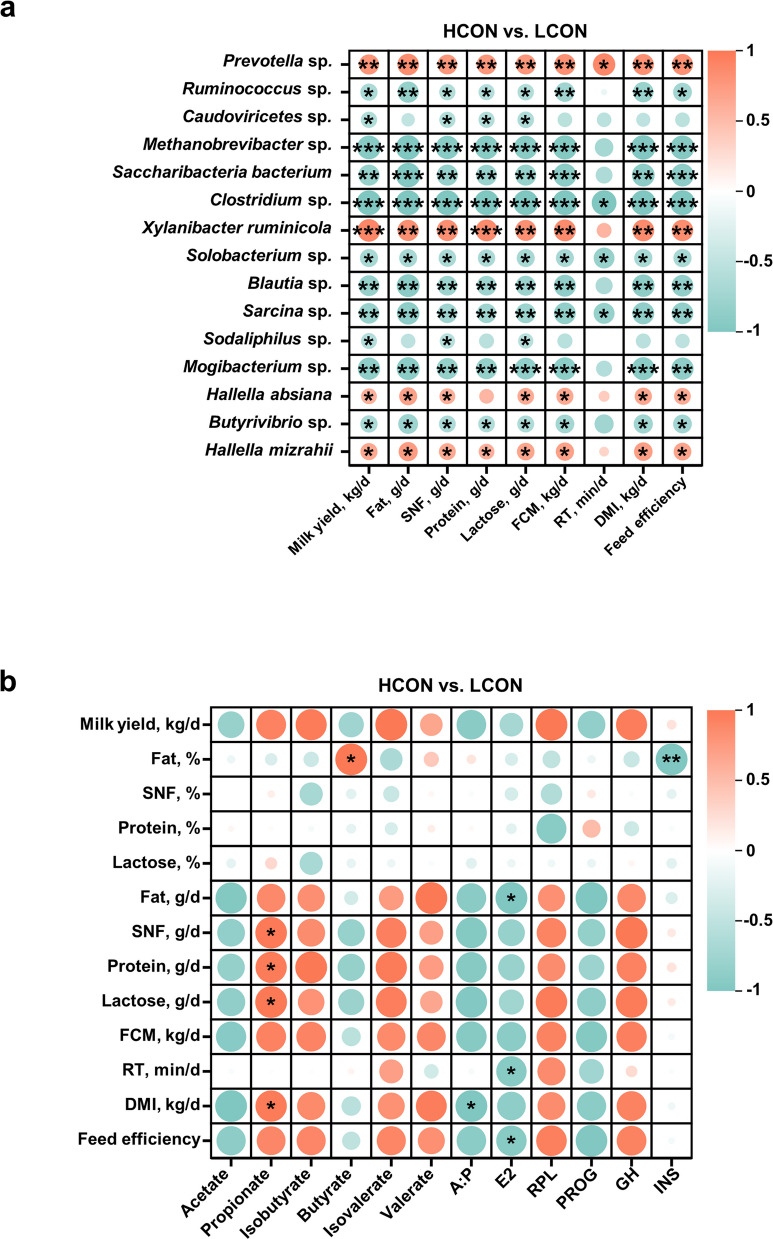
Spearman correlations (|*r*| > 0.5, *P* < 0.05) among lactation performance, differential microbes (species level), rumen fermentation parameters, and circulating hormones in HCON vs. LCON

Spearman’s correlation analysis was subsequently performed to examine the correlations among lactation performance, differential rumen microbes, fermentation parameters, and blood hormones in high- and low-yield cows (Fig. 2). Four species-level microbes enriched in the HCON group (*Prevotella* sp.,* Xylanibacter ruminicola*,* Hallella absiana*, and *Hallella mizrahii*) presented significant positive correlations with lactation performance, whereas 11 species-level microbes enriched in the LCON group (*Ruminococcus* sp*.*,* Caudoviricetes* sp*.*,* Methanobrevibacter* sp*.*,* Saccharibacteria bacterium*,* Clostridium* sp.,* Solobacterium* sp.,* Blautia* sp*.*,* Sarcina* sp.,* Sodaliphilus* sp*.*,* Mogibacterium* sp*.*, and *Butyrivibrio* sp*.*) exhibited significant negative correlations with lactation performance (Fig. 2a, *r* > |0.5|, *P* < 0.05). In the HCON group, four enriched species (*Prevotella* sp.,* Xylanibacter ruminicola*,* Hallella absiana*, and *Hallella mizrahii*) were significantly and positively correlated with DMI and feed efficiency, whereas nine species enriched in the LCON group (*Ruminococcus* sp*.*,* Methanobrevibacter* sp.,* Saccharibacteria bacterium*,* Clostridium* sp.,* Solobacterium* sp*.*,* Blautia* sp*., Sarcina* sp*.*,* Mogibacterium* sp*.*, and *Butyrivibrio* sp*.*) showed significant negative correlations with DMI and feed efficiency (Fig. 2a, *r* > |0.5|, *P* < 0.05). Additionally, *Prevotella* sp. enriched in the HCON group was significantly and positively correlated with RT, whereas *Clostridium* sp., *Solobacterium* sp., and *Sarcina* sp. enriched in the LCON group were significantly and negatively correlated with RT (Fig. 2a, *r* > |0.5|, *P* < 0.05).

Additionally, correlation analysis between lactation performance and rumen fermentation parameters revealed that the proportion of ruminal propionate was significantly positively correlated with milk SNF, protein, lactose, and dry matter intake (Fig. 2b, *r* > |0.5|, *P* < 0.05). The proportion of butyrate was significantly positively correlated with milk fat content. The A:P ratio was significantly negatively correlated with DMI. Blood E2 levels were significantly negatively correlated with milk fat yield, rumination time, and feed efficiency. Blood INS levels were significantly negatively correlated with milk fat content (Fig. 2b, *r* > |0.5|, *P* < 0.05).

### Rumen microbiome structure, function, and phenotype correlations after SIF supplementation in high- and low-yield cows

**Fig. 3:**
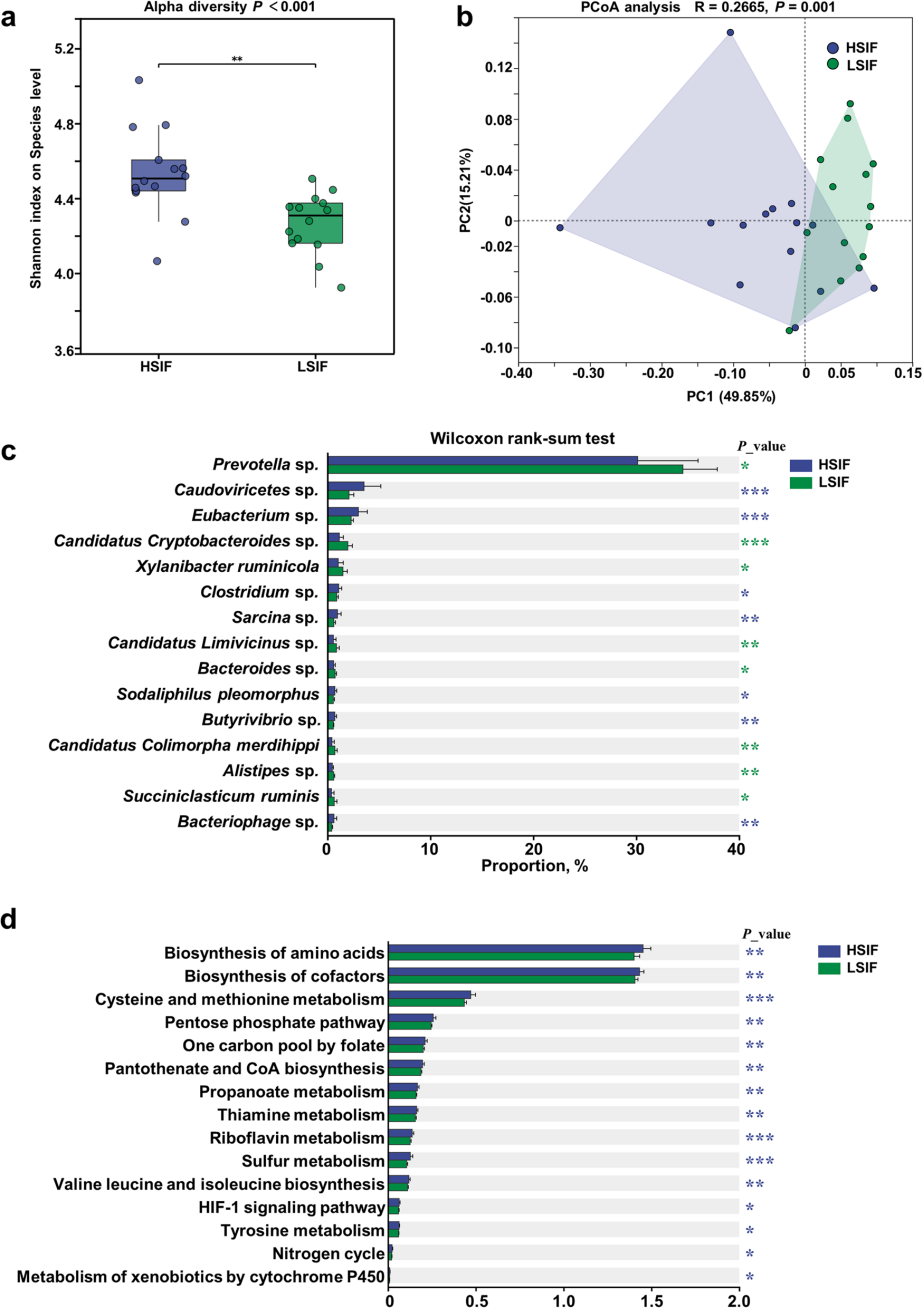
Response of the rumen microbiome of high- and low-yield dairy cows to SIF supplementation (HSIF vs. LSIF). **a** α-diversity (Shannon). **b** PCoA of Bray–Curtis distances (PC1 = 49.85%, PC2 = 15.21%; PERMANOVA *R* = 0.2655, *P* = 0.001). **c** Top 15 differential species (*P* < 0.05). **d** KEGG pathway enrichment (*P* < 0.05)

**Fig. 4:**
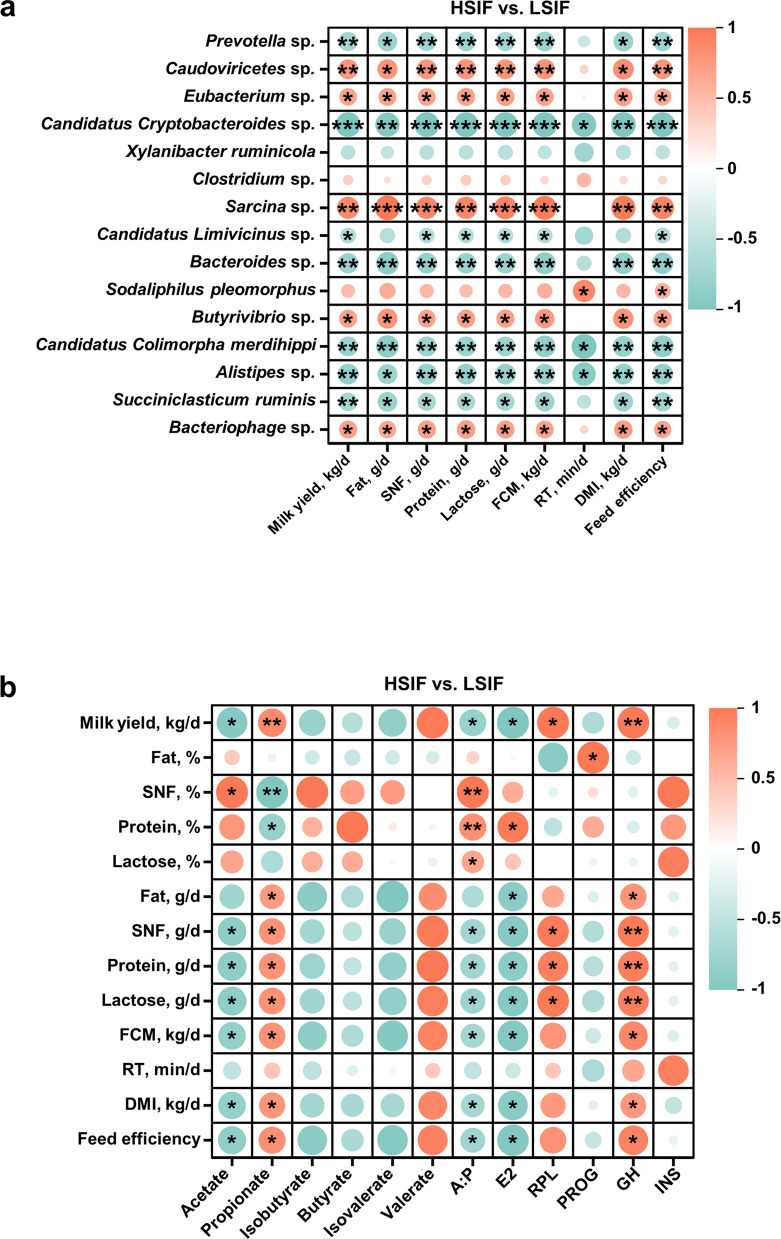
Spearman correlations (|*r*| > 0.5, *P* < 0.05) among lactation performance, differential microbes (species level), rumen fermentation parameters, and circulating hormones in the HSIF vs. LSIF groups

Compared with the LSIF group, the HSIF group presented greater Shannon diversity (Fig. 3a, *P* < 0.001) and distinct β diversity (Fig. 3b, PC1 = 49.85%, PC2 = 15.21%; *R* = 0.2655, *P* = 0.001). *Caudoviricetes* sp.,* Eubacterium* sp.,* Clostridium* sp.,* Sarcina* sp.,* Sodaliphilus pleomorphus*,* Butyrivibrio* sp., and *bacteriophages* were enriched in HSIF group, whereas LSIF presented greater abundances of *Prevotella* sp.,* Candidatus Cryptobacteroides* sp.,* X. ruminicola*,* Candidatus Limivicinus* sp.,* Bacteroides* sp.,* Candidatus Colimorpha merdihippi*,* Alistipes* sp., and *Succiniclasticum ruminis* (relative abundance Top 15; *P* < 0.05, Fig. 3c). KEGG analyses revealed greater capacities of HSIF for the biosynthesis of amino acids, biosynthesis of cofactors, cysteine and methionine metabolism, the pentose phosphate pathway, one-carbon pool by folate, pantothenate and CoA biosynthesis, propanoate metabolism, thiamine metabolism, riboflavin metabolism, sulfur metabolism, valine, leucine and isoleucine biosynthesis, the HIF-1 signalling pathway, tyrosine metabolism, the nitrogen cycle, and metabolism of xenobiotics by cytochrome P450 (*P* < 0.05, Fig. 3d). Correlation analysis revealed that most taxa enriched in the HSIF group were significantly and positively associated with milk production performance, except for *Clostridium* sp. and *Sodaliphilus pleomorphus*, whereas most LSIF-enriched taxa showed significant negative associations, with the exception of *X. ruminicola*. (Fig. 4a, *r* > |0.5|, *P* < 0.05).

Spearman’s correlation analysis was subsequently performed to examine the correlations among lactation performance, differential rumen microbes, fermentation parameters, and blood hormones in high- and low-yield dairy cows supplemented with SIF (Fig. 4). Five species-level microbes enriched in the HSIF group (*Caudoviricetes* sp.,* Eubacterium* spp.,* Sarcina* spp.,* Butyrivibrio* spp., and *Bacteriophage* spp.) showed significant positive correlations with lactation performance, whereas seven species-level microbes enriched in the LSIF group (*Prevotella* sp.,* Candidatus Cryptobacteroides* sp.,* Candidatus Limivicinus* sp*., Bacteroides* sp*.*,* Candidatus Colimorpha merdihippi*,* Alistipes* sp*.*, and *Succiniclasticum ruminis*) presented significant negative correlations with lactation performance (Fig. 4a, *r* > |0.5|, *P* < 0.05). The proportions of ruminal acetate and A:P were significantly negatively correlated with milk yield, milk component yields, DMI, and feed efficiency, whereas the proportion of propionate showed the opposite pattern. Blood E2 levels were significantly negatively correlated with milk component yields, FCM, DMI, and feed efficiency, whereas PRL was significantly positively correlated with milk yield and milk component yields (Fig. 4b, *r* > |0.5|, *P* < 0.05).

### Differential responses of the rumen microbial community structure and function in high- and low-yield dairy cows to SIF supplementation

**Fig. 5:**
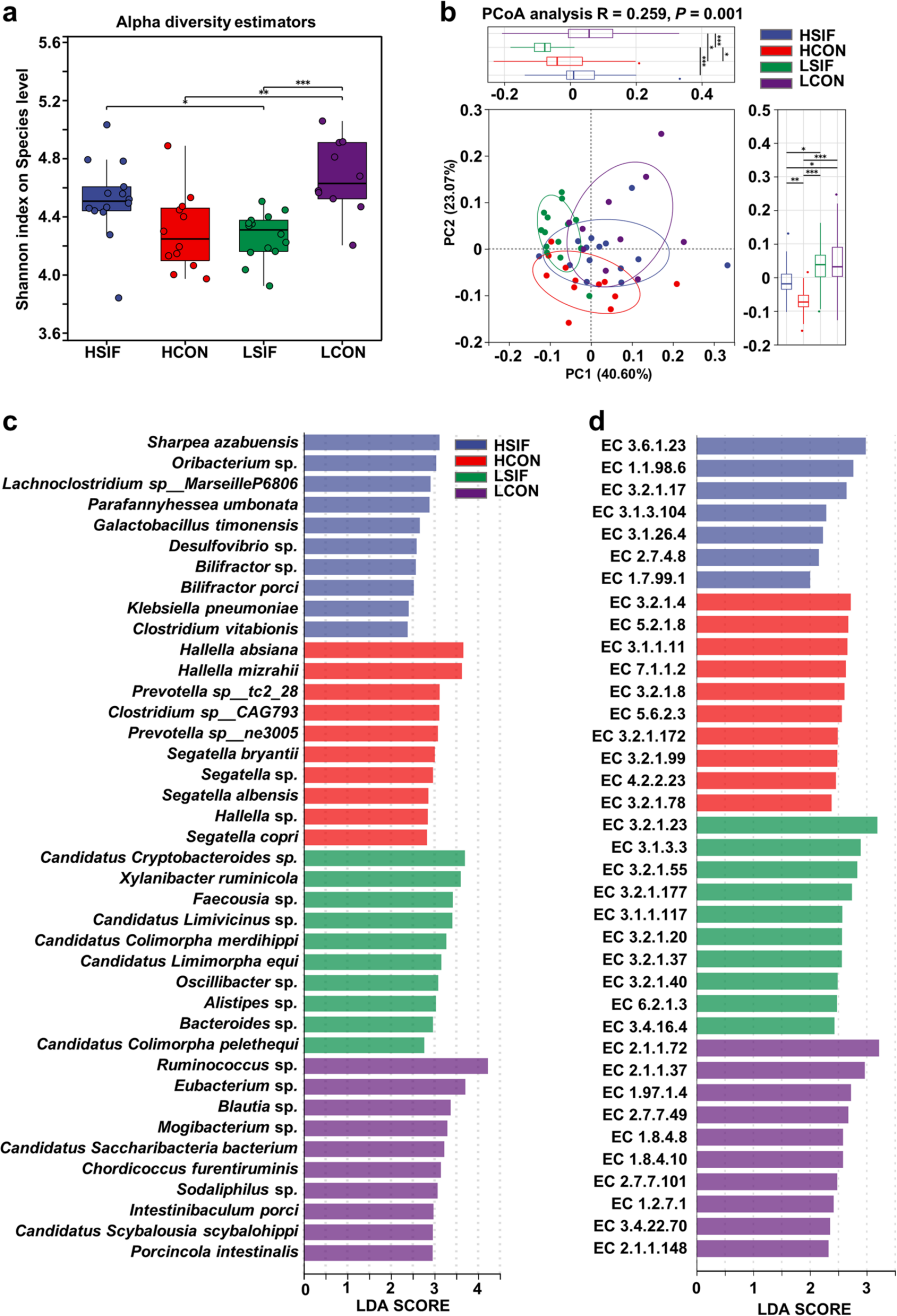
Effects of soybean isoflavones on the rumen microorganisms of dairy cows at the same production level. **a** α-diversity (Shannon; HY: HSIF vs. HCON NS; LY: LSIF vs. LCON decreased). **b** β-diversity (Bray–Curtis; *P* < 0.05). **c** LEfSe differential species (LDA > 2, *P* < 0.05). **d** Differential KEGG enzyme (EC) profiles (*P* < 0.05)

The differences in the rumen microbial composition and functions between high-yield and low-yield cows, with or without SIF supplementation, were further identified (Fig. 5). SIF did not affect α-diversity in high-yield cows (HSIF vs. HCON, *P* > 0.05) but reduced Shannon diversity in low-yield cows (Fig. 5a, LSIF vs. LCON, *P* < 0.05), whereas β-diversity shifted in both strata (Fig. 5b, *P* < 0.05).

In terms of differential microbial species, the LEfSe analysis showed that *Sharpea azabuensis*,* Oribacterium* sp*.*,* Lachnoclostridium* sp. *Marseille P6806*,* Parafannyhessea umbonate*,* Galactobacillus timonensis*,* Desulfovibrio* sp.,* Bilifractor* sp.,* Bilifractor porci*,* Klebsiella pneumoniae* and *Clostridium vitabionis* were significantly enriched in the HSIF group; *Hallella absiana*,* Hallella mizrahii*,* Prevotella* sp. tc2_28,* Clostridium* sp. CAG793,* Prevotella* sp. *ne3005*,* Segatella bryantii*, *Segatella* sp., *Segatella albensis*, *Hallella* sp., and *Segatella copri* were significantly enriched in the HCON group; *Candidatus Cryptobacteroides* sp., *Xylanibacter ruminicola*, *Faecousia* sp., *Candidatus Limivicinus* sp., *Candidatus Colimorpha merdihippi*, *Candidatus Limimorpha equi*, *Oscillibacter* sp., *Alistipes* sp., *Bacteroides* sp., and *Candidatus Colimorpha pelethequiwere* significantly enriched in the LSIF group; while *Ruminococcus* sp., *Eubacterium* sp., *Blautia* sp., *Mogibacterium* sp., *Candidatus Saccharibacteria bacterium*, *Chordicoccus furentiruminis*, *Sodaliphilus* sp., *Intestinibaculum porci*, *Candidatus Scybalousia scybalohippi*, and *Porcincola intestinalis* were significantly enriched in the LCON group (Fig. 5c, LDA > 2, *P* < 0.05).

Functional profiling of differentially abundant taxa indicated group-specific metabolic potentials (Fig. 5d, LDA > 2, *P* < 0.05). The HSIF group exhibited enrichment in enzymes linked to nucleotide metabolism and cell wall turnover, including dUTP diphosphatase (EC 3.6.1.23), ribonucleoside-triphosphate reductase (EC 1.1.98.6), ribonuclease H (EC 3.1.26.4), guanylate kinase (EC 2.7.4.8), and lysozyme (EC 3.2.1.17). In HCON, carbohydrate-active enzymes dominated, such as cellulase (EC 3.2.1.4), endo-1,4-β-xylanase (EC 3.2.1.8), pectinesterase (EC 3.1.1.11), and arabinosidases (EC 3.2.1.99, EC 3.2.1.55), suggesting enhanced dietary fiber degradation. The LSIF group was enriched in enzymes involved in carbohydrate metabolism, including β-galactosidase (EC 3.2.1.23), α-glucosidase (EC 3.2.1.20), and serine-type D-Ala-D-Ala carboxypeptidase (EC 3.4.16.4). Conversely, the LCON group showed enrichment in enzymes associated with DNA modification and energy metabolism, including DNA methyltransferases (EC 2.1.1.37, EC 2.1.1.72), pyruvate formate-lyase activating enzyme (EC 1.97.1.4), pyruvate synthase (EC 1.2.7.1), and sortase A (EC 3.4.22.70).

**Fig. 6:**
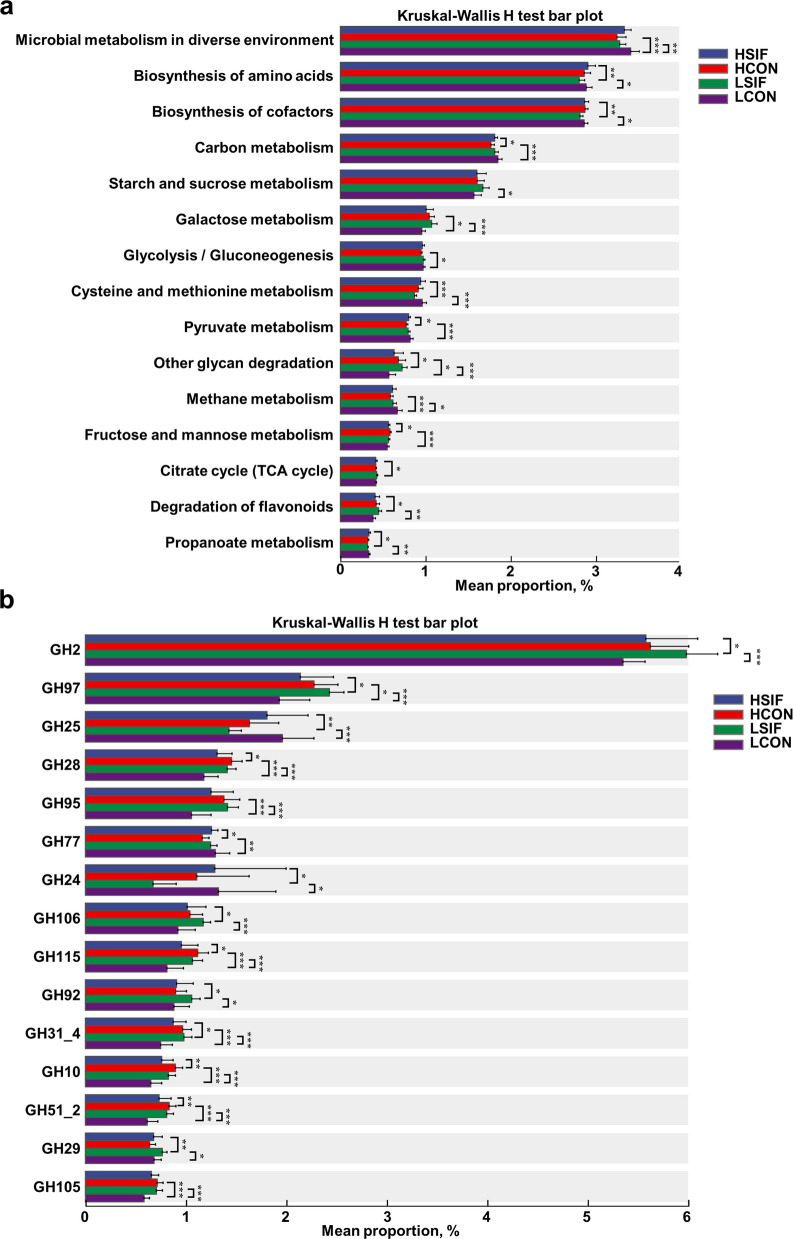
Ruminal function shifts after SIF supplementation. **a** KEGG pathway enrichment (HCON vs. LCON, HSIF vs. LSIF, HCON vs. HSIF, LCON vs. LSIF, *P* < 0.05). **b** CAZ enrichment (HCON vs. LCON, HSIF vs. LSIF, HCON vs. HSIF, LCON vs. LSIF, *P* < 0.05)

KEGG enrichment analysis revealed significant functional remodeling of the rumen microbiome under different milk production levels and in response to SIF supplementation (Fig. 6a). Compared with LCON, the HCON group was enriched in pathways related to carbohydrate utilization, including other glycan degradation and fructose and mannose metabolism, whereas LCON exhibited significant enrichment of central carbon and methane-associated pathways, including microbial metabolism in diverse environments, carbon metabolism, glycolysis/gluconeogenesis, pyruvate metabolism, and methane metabolism (*P* < 0.05). This indicates that the rumen metabolic characteristics of low-yield cows are biased towards extensive carbon transformation and methane generation, whereas high-yield cows focus on the capacity for carbohydrate degradation. Compared with LSIF, the HSIF microbiome was significantly enriched in energy metabolism pathways, including biosynthesis of amino acids, biosynthesis of cofactors, cysteine and methionine metabolism, the citrate cycle (TCA cycle), and propanoate metabolism (*P* < 0.05). In contrast, LSIF were enriched in other glycan degradation and flavonoid degradation (*P* < 0.05), indicating a stronger capacity for substrate decomposition but a limited ability in energy metabolism transformation. In high-yield cows, SIF supplementation (HSIF vs. HCON) enhanced carbon metabolism and pyruvate metabolism, whereas fructose and mannose metabolism were more abundant in HCON (*P* < 0.05), indicating that SIF shifts primary sugar degradation towards energy production and metabolic processes. In low-yield cows, SIF supplementation (LSIF vs. LCON) resulted in significant enrichment of carbohydrate metabolism pathways (starch and sucrose metabolism, galactose metabolism, other glycan degradation and propanoate metabolism), whereas LCON remained enriched in microbial metabolism in diverse environments, amino acid and cofactor biosynthesis, and notably methane metabolism (*P* < 0.05). These findings suggest that the supplementation of SIF can to some extent shift low-yield cows from a methane-producing and basal metabolic state to a high-efficiency carbohydrate metabolism state dominated by propionic acid, although the functional remodeling is not as obvious as that in high-yield cows. CAZyme enrichment analysis revealed that GH25, GH77, and GH24 were significantly increased in the HSIF group, whereas GH2, GH97, GH28, GH95, GH106, GH115, GH92, GH31_4, GH10, GH51_2, GH29, and GH105 were significantly increased in the LSIF group (Fig. 6b, *P* < 0.05). Collectively, these data indicate that SIF induced a yield-dependent remodeling of the rumen microbial metabolic network, reflecting the promotion of anabolic capacity and propionate metabolism in high-yield cows, while diminishing the prominence of methane-related pathways.

### Differences in the metabolomic profiles of rumen in high- and low-yield cows

**Fig. 7:**
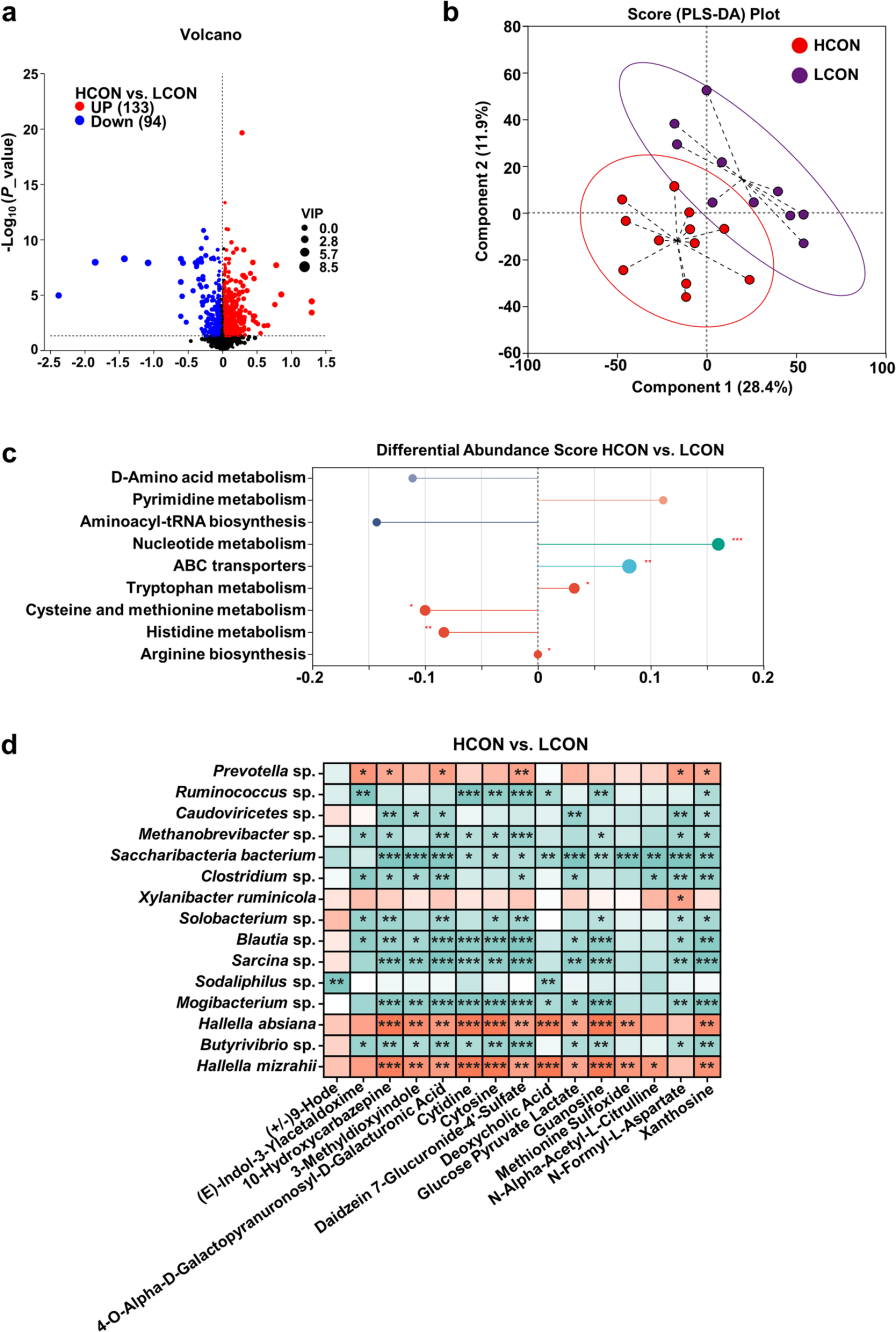
Baseline rumen metabolome (HY-CON vs. LY-CON). **a** Differentially abundant metabolites (VIP > 1, *P* < 0.05). **b** PLS-DA score plot. **c** KEGG pathway enrichment (top 15 by fold change; *P* < 0.05). **d** Microbe–metabolite Spearman correlations (|*r*| > 0.5, *P* < 0.05)

Metabolomic analysis of dairy cow rumen fluid revealed (HCON vs. LCON) that 133 metabolites were significantly upregulated and 94 metabolites were significantly downregulated (Fig. 7a, VIP > 1, *P* < 0.05). The results of partial least squares discriminant analysis (PLS-DA) revealed a significant separation between the HCON group and the LCON group, suggesting a significant difference in metabolic status between the two groups (Fig. 7b). KEGG enrichment analysis of the differentially abundant metabolites in the rumen revealed that the pathways associated with nucleotide metabolism, ABC transporters, tryptophan metabolism, and arginine biosynthesis were significantly enriched in the HCON group, whereas the pathways associated with cysteine and methionine metabolism and histidine metabolism were significantly enriched in the LCON group (Fig. 7c; VIP > 1, *P* < 0.05; FC: TOP 15). Furthermore, Spearman correlation analysis of the differential microbes and metabolites in the rumen from the HCON and LCON groups was conducted. The significantly enriched microbes in the HCON group (*Prevotella* sp*.*,* Hallella absiana*, and *Hallella mizrahii*) presented significant positive correlations with the upregulated differentially abundant metabolites ((E)-Indol-3-Ylacetaldoxime, 10-Hydroxycarbazepine, 3-Methyldioxyindole, etc.), whereas the significantly enriched microbes in the LCON group presented significant negative correlations with the aforementioned differentially abundant metabolites (Fig. 7d, *r* > |0.5|, *P* < 0.05).

### Effects of SIF supplementation on the ruminal metabolomic profiles of high- and low-yield cows

**Fig. 8:**
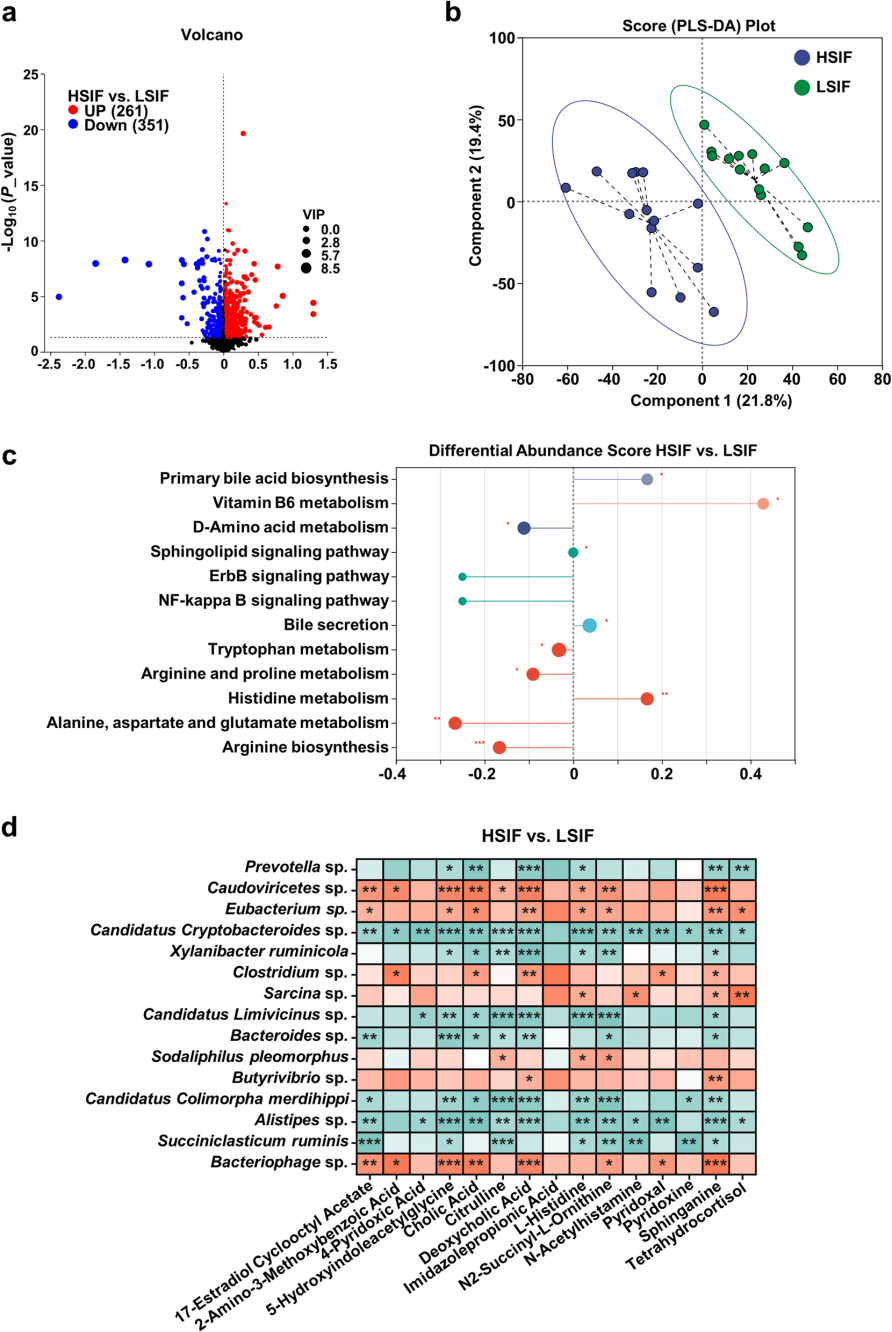
Response of the rumen metabolome of high- and low-yielding dairy cows to SIF supplementation (HSIF vs. LSIF). **a** Differentially abundant metabolites (VIP > 1, *P* < 0.05). **b** PLS-DA score plot. **c** KEGG pathway enrichment (top 15 by fold change; *P* < 0.05). **d** Microbe–metabolite Spearman correlations (|*r*| > 0.5, *P* < 0.05)

Further investigation was conducted to determine the differential effects of SIF supplementation on the ruminal metabolomic profiles of high- and low-yield dairy cows (Fig. 8). Compared with those in the LSIF group, 261 metabolites in the HSIF group were significantly upregulated, and 351 metabolites were significantly downregulated (Fig. 8a, VIP > 1, *P* value < 0.05). The PLS-DA results indicated a significant separation between the HSIF group and the LSIF group, suggesting a significant difference in metabolic status between the two groups (Fig. 8b). KEGG enrichment analysis of the differentially abundant metabolites in the rumen revealed that the pathways of primary bile acid biosynthesis, vitamin B_6_ metabolism, bile secretion, and histidine metabolism were significantly enriched in the HSIF group, whereas the pathways of D-amino acid metabolism, tryptophan metabolism, arginine and proline metabolism, alanine, aspartate and glutamate metabolism, and arginine biosynthesis were significantly enriched in the LSIF group (Fig. 8c, VIP > 1, *P* < 0.05, FC: TOP 15). Moreover, a Spearman correlation analysis of the differential microbes and metabolites in the rumen from the HSIF and LSIF groups was conducted. The significantly enriched microbes in the HSIF group (*Caudoviricetes* spp.,* Eubacterium* spp.,* Clostridium* spp.,* Sarcina* spp.,* Sodaliphilus pleomorphus*,* Butyrivibrio* spp., and *Bacteriophage* spp.) showed significant positive correlations with the upregulated differentially abundant metabolites (17-Estradiol Cyclooctyl Acetate, 2-Amino-3-Methoxybenzoic Acid, 4-Pyridoxic Acid, 5-Hydroxyindoleacetylglycine, etc.), whereas the significantly enriched microbes in the LSIF group presented significant negative correlations with the aforementioned differentially abundant metabolites (Fig. 8d, *r* > |0.5|, *P* < 0.05).

**Fig. 9:**
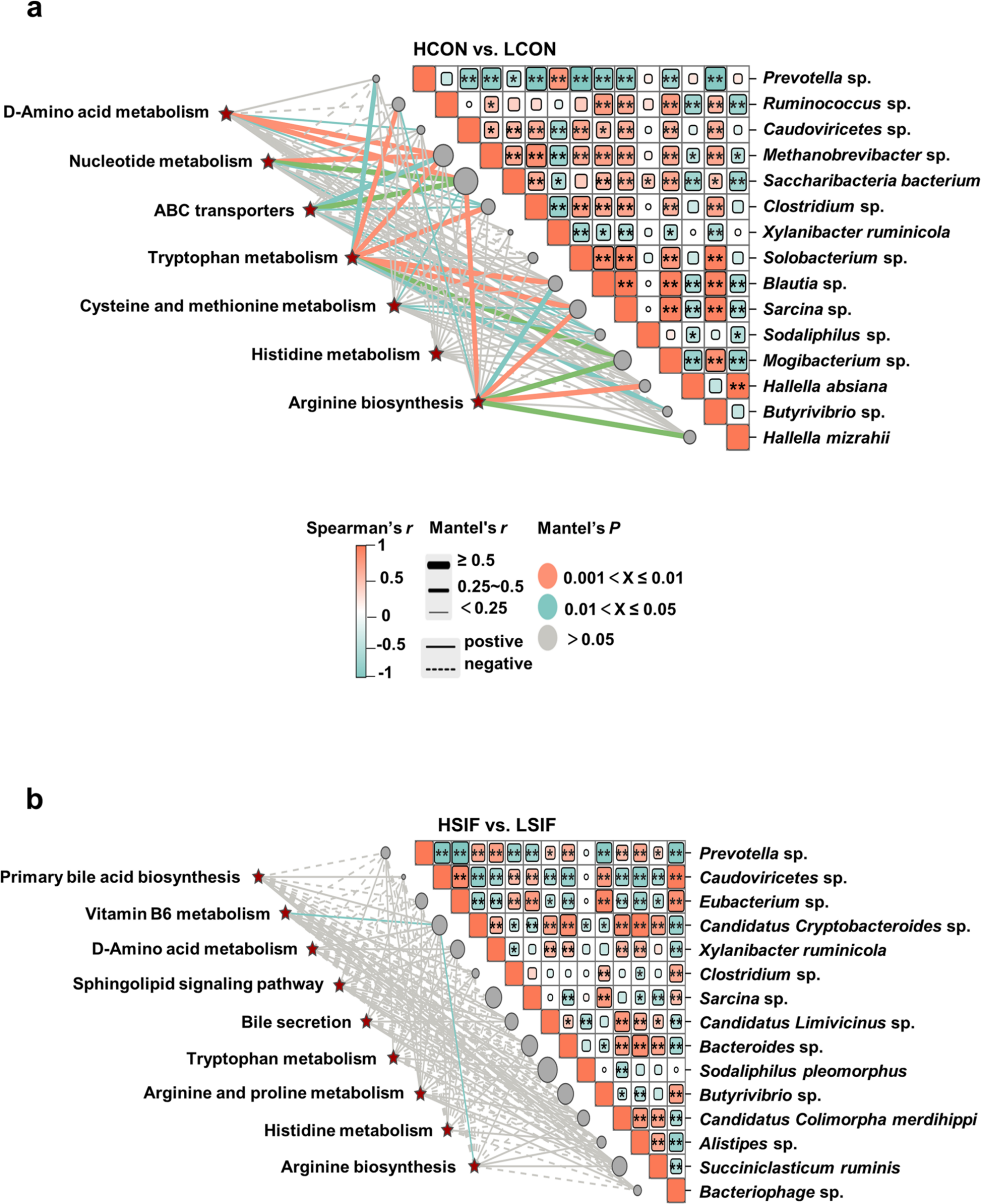
Mantel tests linking differential microbial matrices and KEGG pathways enriched by differentially abundant metabolites (Euclidean distances; |*r*| > 0.5, *P* < 0.05)

To further confirm the interactions between rumen microbes and metabolites, we used the Mantel test to associate the matrix of differential microbial composition with the KEGG pathways enriched with differentially abundant metabolites (Fig. 9). In both high- and low-yield dairy cows, *Prevotella* sp*.* was significantly positively correlated with tryptophan metabolism; *Ruminococcus* sp*.* showed significant positive correlations with tryptophan metabolism and arginine biosynthesis; *Caudoviricetes* sp*.* was significantly positively correlated with D-amino acid metabolism; *Methanobrevibacter* sp*.* showed significant positive correlations with D-amino acid metabolism, nucleotide metabolism, ABC transporters, and tryptophan metabolism; *Saccharibacteria bacterium* was significantly positively correlated with ABC transporters, cysteine and methionine metabolism, and arginine biosynthesis; *Clostridium* sp*.* had significant positive correlations with D-amino acid metabolism, nucleotide metabolism, ABC transporters, and tryptophan metabolism; *Blautia* sp*.* showed significant positive correlations with tryptophan metabolism and arginine biosynthesis; *Sarcina* sp*.* was significantly positively correlated with tryptophan metabolism, cysteine and methionine metabolism, and arginine biosynthesis; *Sodaliphilus* sp*.* showed significant positive correlations with nucleotide metabolism, ABC transporters, and cysteine and methionine metabolism; *Mogibacterium* sp*.* was significantly positively correlated with tryptophan metabolism and arginine biosynthesis; *Hallella absiana* was significantly positively correlated with arginine biosynthesis; *Butyrivibrio* sp*.* was significantly positively correlated with tryptophan metabolism, and *Hallella mizrahii* was significantly positively correlated with arginine biosynthesis (Fig. 9a, *r* > |0.5|, *P* < 0.05). After SIF supplementation, *Candidatus Cryptobacteroides* sp*.* was significantly positively correlated with vitamin B_6_ metabolism and arginine biosynthesis (Fig. 9b, *r* > |0.5|, *P* < 0.05).

## Discussion

This study verified the hypothesis that dietary supplementation with SIF alters rumen function and host physiology in dairy cows, as well as the different responses to SIF in high- and low-yield dairy cows. Briefly, SIF improved milk and component yields alongside a shift toward a higher rumen fermentation efficiency (higher propionate, lower A:P, and greater MCP), and reshaped the endocrine status (increased E2 and PROG and decreased PRL and GH), and restructured the rumen microbiome and its functional repertoire. These findings provide important theoretical evidence for precision nutrition strategies, integrating multiomics data to identify microbial-metabolic-host pathways supporting SIF efficacy, emphasizing the importance of individualized feeding strategies for improving dairy cow productivity.

This study demonstrates that supplementation of SIF at 0.01% of dietary (DM) in early‑lactation Holstein cows significantly increased milk yield. SIF remodeled rumen metabolism around a central network of “SIF-fermentation shift-increased MCP synthesis- improved feed efficiency” with more pronounced responses in high‑yielding cows. Overall, SIF increased DMI by only 3.20% while milk yield rose by 8.75%, corresponding to a 3.26% increase in feed efficiency. These results indicate that the improvement in feed efficiency was not solely driven by increased intake, but rather by enhanced usable energy and utilizable protein per unit of feed. This effect was more pronounced in high‑yielding cows: DMI increased by 3.63%, whereas milk yield and FCM increased by 8.89% and 8.92%, respectively, resulting in a 4.55% improvement in feed efficiency.

At the rumen fermentation level, SIF increased the propionate proportion by 4.55% and decreased the A:P ratio by 7.02%, while markedly increasing MCP by 21.53%. These fermentation changes are consistent with improved gluconeogenic substrate supply and microbial protein synthesis. Supporting evidence from a study of a multi‑strain inoculant (*Lactobacillus plantarum, L. buchneri, Propionibacterium acidipropionici, P. thoeni*) shows that higher ruminal propionate, lower methane emission intensity, and greater nutrient digestibility are closely associated with improved feed efficiency in dairy cows [5]. Improved feed efficiency therefore not only reduces feed costs per unit of milk but also lowers greenhouse‑gas emission intensity per unit of product. Notably, NH₃‑N concentrations did not change while MCP increased significantly, suggesting improved nitrogen utilization efficiency rather than increased ammonia production. These results align with previous studies reporting that isoflavone-enriched diets regulate rumen fermentation and improve the antioxidant and immune status of dairy cows [37, 49]. Previous studies have shown that daidzein can increase the yield of propionate in the in vitro rumen fermentation broth of goats, and the yield of propionate is the highest when the content of daidzein is 10 mg/L [29]. Propionate infusion has been shown to increase gluconeogenesis and increase the glucose supply, thus increasing milk protein production [50–53]. Propionic acid is the main carbon source for gluconeogenesis in the liver of ruminants. Theoretically, increasing the supply of propionic acid is closely related to lactose synthesis and the improvement of milk production [53]. In this study, an increase in plasma glucose levels was observed in the high-yield group, and propionate was positively correlated with SNF, protein, and lactose in milk, supporting this gluconeogenic mechanism. The increase in MCP further suggests enhanced microbial growth and nitrogen use efficiency, which is consistent with the role of isoflavones as redox-active regulators that promote bacterial proliferation and protein synthesis [37]. Therefore, the observed improvement in feed efficiency in this study is best explained by a synergistic mechanism whereby enhanced energy supply efficiency (increased ruminal propionate) and greater metabolizable protein availability (elevated MCP) contribute to increased milk production.

For the first time, this study systematically compared the blood hormone response patterns of high- and low-yield dairy cows following SIF supplementation. The results showed that SIF significantly increased E2 and PROG concentrations while decreasing PRL and GH levels, with no impact on INS. Notably, the trends of these hormonal changes were consistent across different milk production levels, suggesting that the estrogen-like activity of SIF [19] in regulating endocrine function in dairy cows is independent of milk yield. As a plant-derived phytoestrogen, SIF has a structure similar to that of 17β-estradiol and can bind to estrogen receptors (ERα and ERβ), regulating downstream signaling pathways [54]. In a study of mice supplemented with isoflavones for 6 weeks, increases in blood E2 and PROG levels were observed [55], which is consistent with the endocrine results noted in this study. Conversely, the decreased levels of PRL and GH indicate feedback inhibition at the hypothalamic‒pituitary level. One study revealed that PROG can inhibit the expression of PRL in the anterior pituitary of adult rats [56], which may explain the changes in PROG and PRL levels in the blood of dairy cows following SIF supplementation. High levels of E2 have been shown to suppress GH secretion [57], and the decline in GH levels in both high- and low-yield cows may reflect a shift in nutritional allocation from growth promotion to activation of the reproductive axis, in line with the increased PROG concentration, highlighting the potential benefits of SIF on dairy cow reproductive performance. Additionally, the reduction in ALP, LAC, and TG in the blood indicates improved liver function and lipid metabolism, which is consistent with the antioxidant and anti-inflammatory properties of isoflavones [37, 49].

At the microbial level, we identified *Prevotella* sp*.*,* Xylanibacter ruminicola*,* Hallella absiana*, and *Hallella mizrahii* as high-yield associated microbial species, whereas *Ruminococcus* sp*.*,* Caudoviricetes* sp*.*,* Methanobrevibacter* sp*.*,* Saccharibacteria bacterium*,* Clostridium* sp*.*,* Solobacterium* sp*.*,* Blautia* sp*.*,* Sarcina* sp*.*,* Sodaliphilus* sp*.*,* Mogibacterium* sp*.*, and *Butyrivibrio* sp*.* were linked to low-yield cows. This aligns with findings in larger studies that reported that the abundance of Bacteroidetes and propionate-producing communities was associated with higher efficiency, supporting the idea that both the host and microbiome jointly determine feed efficiency [8, 58–61].

Significant differences in α and β diversity, species composition, and functionality between the high- and low-yield groups indicate that the rumen microbiota is markedly distinct between these groups, which may influence the SIF utilization efficiency. In the control group, high-yield cows were enriched with microbial communities significantly positively correlated with lactation performance (e.g., *Prevotella* sp*.*,* Hallella absiana*, * Hallella mizrahii*), whereas low-yield cows presented relatively high relative abundances of *Ruminococcus* sp*.*,* Mogibacterium* sp*.*,* and Methanobrevibacter* sp*.* Rumen microbiota are primarily composed of *Prevotella* sp*.* is typically associated with efficient carbohydrate degradation and propionate-type fermentation metabolism [8, 62, 63], whereas communities rich in *Ruminococcus* sp. and *Methanobrevibacter* sp. tends to produce acetate and hydrogen, promoting methane generation and reducing feed efficiency [34, 61].

After SIF treatment, the high-yield dairy cow rumen was enriched with microbes such as *Caudoviricetes* sp*.*,* Eubacterium* sp*.*,* Sarcina* sp*.*,* Sodaliphilus pleomorphus*,* Butyrivibrio* sp*.*, and *Bacteriophage* sp*.*, whose functional advantages include amino acid, cofactor (vitamin B_6_, thiamine, riboflavin), and propionate metabolism. These changes enhanced the biosynthetic capacity of the microbes and improved energy efficiency along the propionate pathway, supporting better energy and nitrogen utilization in the rumen. In contrast, low-yield dairy cows enriched *Prevotella* sp*.*, *Bacteroides* sp*.*, and *Succiniclasticum ruminis* after-SIF treatment, with a focus on starch and sucrose metabolism, galactose metabolism, and other carbohydrate degradation. Although these pathways support energy generation, their nitrogen cycling capabilities, such as amino acid synthesis and cofactor metabolism, are weaker, indicating that the lower production performance in low-yield dairy cows may be related to the mismatch in energy and nitrogen utilization rates.

The biological activity of SIF depends on microbial deglycosylation and subsequent reduction to generate bioactive aglycone metabolites similar to equol [18, 64]. The rumen microbiota can effectively cleave and reduce isoflavones [30], but the amount of equol and related metabolites produced is influenced by community composition and enzymes [65–67]. After SIF treatment, enzymes involved in isoflavonoid degradation (GH2, GH29, GH95, GH97, cellulase, mannan, endo-1,4-beta-mannosidase, beta-galactosidase, etc.), polysaccharide-degrading microbial communities (e.g., *Clostridium vitabionis*,* Oribacterium* sp*.*,* Bilifractor* sp*.*,* Faecousia* sp*.*,* Bacteroides* sp*.*), and a greater flavonoid degradation function (degradation of flavonoids) were significantly enriched, facilitating the rapid activation of SIF. Furthermore, the metabolomic results revealed significant increases in metabolites such as 2,4-bis(4-hydroxybenzyl)phenol, flavonol 3-O-D-glucoside, and daidzein 7-O-glucuronide, further confirming the role of rumen microbes in isoflavone degradation.

Although we integrated multiomics data, we could still not determine causal relationships; inferences were made through pathway enrichment rather than direct quantification. Future studies will focus on combining *Prevotella* sp. or β-glucosidase-positive probiotics to test their synergistic effects on propionate and MCP, as well as utilizing meta-transcriptomics and molecular biology to explore the mechanisms by which SIF regulate differences in milk yield responses via the rumen‒liver‒mammary gland axis. Although this study found consistent endocrine regulation of SIF (soy isoflavones) in both high- and low-yield cows, its effects on rumen metabolism and microbiome composition exhibited milk yield-dependent differences. Our current data cannot explain this phenomenon. In the future, we aim to assess the bioavailability of SIF and active metabolites (concentration dynamics) or tissue sensitivity differences (in the rumen, liver, gonads, mammary glands, etc.) in cows with varying milk yields. This will enable establishing a cascading network linking host physiological status, rumen microecology, SIF metabolism, and endocrine responses, ultimately helping to understand the effects of functional additives under different production scenarios.

**Fig. 10:**
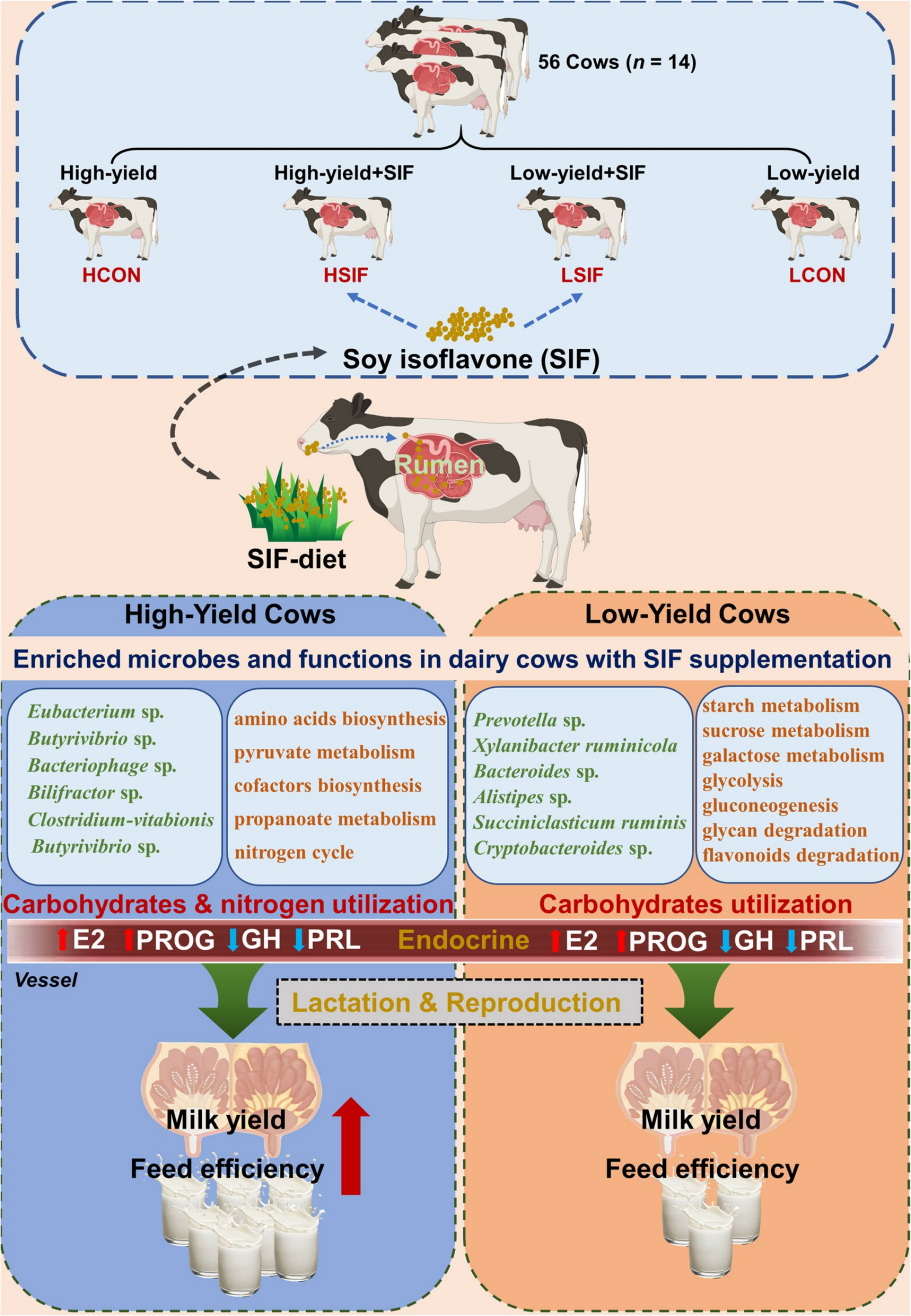
Effects of soybean isoflavones (SIF) on lactation performance, rumen fermentation, microbiota structure and function, and endocrine regulation in dairy cows. (1) SIF supplementation decreased the ruminal acetate proportion and A:P ratio while increasing propionate and MCP concentrations, shifting rumen fermentation toward a more efficient propionate-dominant profile. (2) SIF significantly altered the rumen microbiome; HSIF enriched beneficial isoflavone-degrading taxa, including *Eubacterium* sp.,* Butyrivibrio* sp.,* Bacteriophage* sp.*, Bilifractor* sp., *Clostridium vitabionis*, and *Butyrivibrio* sp., and enhanced pathways related to amino acid biosynthesis, propanoate metabolism, and pyruvate metabolism, leading to a more balanced utilization of energy and protein. (3) SIF increased plasma E2 and PROG levels, potentially benefiting reproductive performance, while reducing PRL and GH levels, which could help redirect energy towards lactation rather than nonlactational tissues. These endocrine changes were consistent across high- and low-yield cows, suggesting that SIF may optimize hormonal homeostasis to increase lactation efficiency

As illustrated in the overall schematic summary of this study (Fig. 10), our research demonstrated that SIF enhances lactation performance in dairy cows, leading to a shift towards efficient propionate-type fermentation, accompanied by endocrine and rumen microbiome–metabolome remodeling. These effects are more pronounced in high-yield dairy cows, providing a theoretical basis for the application of SIF as a novel functional additive in the precise nutritional regulation of the dairy cow microbiome.

## Conclusion

Supplementation with SIF at dose of 0.01% DM improved lactation performance, increasing milk yield by 8.75% (with component yields: fat + 6.12%, SNF + 8.03%, protein + 7.62%, lactose + 7.84%) and raising FCM by 7.20% alongside DMI + 3.20% and feed efficiency + 3.26%. SIF shifted fermentation toward a more efficient pattern, with propionate molar proportion + 4.55%, acetate − 2.70%, A:P − 7.02%, and MCP + 21.53% (including + 24.25% in high-yield and + 18.88% in low-yield cows) reshaping the endocrine profile and rumen microbiome. These responses were more pronounced in high-yield cows, where milk yield increased 8.89% and feed efficiency improved 4.55%. This study provides theoretical support for the use of SIF as a novel functional additive in precision microbe-based nutrition strategies for dairy cows.

## Data Availability

The data produced or analysed during the current study are available from the corresponding author upon reasonable request.

## Abbreviations

A:P: The ratio of acetate to propionate
ADF: Acid detergent fibre
ALB: Albumin
ALP: Alkaline phosphatase
ALT: Alanine aminotransferase
AST: Aspartate aminotransferase
ATP: Adenosine triphosphate
CHO: Cholesterol
CP: Crude protein
DM: Dry matter
DMI: Dry matter intake
E2: Estradiol
FCM: Fat-corrected milk yield
FDR: False discovery rate
GH: Growth hormone
GLU: Glucose
HY: High-yield
INS: Insulin
LAC: Lactic acid
LDH: Lactate dehydrogenase
LEfSe: Linear discriminant analysis effect size
LY: Low-yield
MCP: Microbial crude protein
NDF: Neutral detergent fibre
OPLS-DA: Orthogonal partial least squares discriminant analysis
PRL: Prolactin
PROG: Progesterone
SIF: Soybean isoflavones
SNF: Solids-not-fat
SOD: Superoxide dismutase
TBA: Total bile acid
TG: Triglyceride
TP: Total protein
UREA: Urea nitrogen
VFA: Volatile fatty acids
VIP: Variable importance in projection
